# Antioxidants, minerals and vitamins in relation to Crohn's disease and ulcerative colitis: A Mendelian randomization study

**DOI:** 10.1111/apt.17392

**Published:** 2023-01-16

**Authors:** Jie Chen, Xixian Ruan, Shuai Yuan, Minzi Deng, Han Zhang, Jing Sun, Lili Yu, Jack Satsangi, Susanna C. Larsson, Evropi Therdoratou, Xiaoyan Wang, Xue Li

**Affiliations:** ^1^ Department of Gastroenterology The Third Xiangya Hospital, Central South University Changsha China; ^2^ Centre for Global Health Zhejiang University School of Medicine Hangzhou China; ^3^ Department of Big Data in Health Science School of Public Health and The Second Affiliated Hospital, Zhejiang University School of Medicine Hangzhou Zhejiang China; ^4^ Unit of Cardiovascular and Nutritional Epidemiology Institute of Environmental Medicine, Karolinska Institutet Stockholm Sweden; ^5^ Translational Gastroenterology Unit, Nuffield Department of Medicine, Experimental Medicine Division University of Oxford, John Radcliffe Hospital Oxford UK; ^6^ Unit of Medical Epidemiology, Department of Surgical Sciences Uppsala University Uppsala Sweden; ^7^ Centre for Global Health Usher Institute, University of Edinburgh Edinburgh UK; ^8^ Cancer Research UK Edinburgh Centre Medical Research Council Institute of Genetics and Cancer, University of Edinburgh Edinburgh UK

**Keywords:** antioxidants, Crohn’s disease, Mendelian randomization analysis, minerals, ulcerative colitis, vitamins

## Abstract

**Background:**

Evidence for antioxidants, minerals and vitamins in relation to the risk of Crohn's disease (CD) and ulcerative colitis (UC) is limited and inconsistent. This mendelian randomization (MR) study aimed to examine the causal associations of circulating levels of antioxidants, minerals and vitamins with CD and UC.

**Methods:**

Single‐nucleotide polymorphisms associated with antioxidants (beta‐carotene, lycopene and uric acid), minerals (copper, calcium, iron, magnesium, phosphorus, zinc and selenium), and vitamins (folate, vitamins A, B6, B12, C, D, E and K1) were employed as instrumental variables. Genetic associations with CD and UC were extracted from the UK Biobank, the FinnGen study and the International Inflammatory Bowel Disease Genetics Consortium. The inverse variance weighted method and sensitivity analyses were performed.

**Results:**

Genetically predicted higher lycopene (OR = 0.94, 95% CI: 0.91–0.97), vitamins D (OR = 0.65, 95% CI: 0.54–0.79) and K1 (OR = 0.93, 95% CI: 0.90–0.97) levels were inversely associated with CD risk, whereas genetically predicted higher magnesium (OR = 1.53, 95% CI: 1.23–1.90) levels were positively associated with CD risk. Higher levels of genetically predicted lycopene (OR = 0.91, 95% CI: 0.88–0.95), phosphorus (OR = 0.69, 95% CI: 0.58–0.82), selenium (OR = 0.91, 95% CI: 0.85–0.97), zinc (OR = 0.91, 95% CI: 0.89–0.94), folate (OR = 0.71, 95% CI: 0.56–0.92) and vitamin E (OR = 0.78, 95% CI: 0.69–0.88) were associated with reduced UC risk, whereas genetically predicted high levels of calcium (OR = 1.46, 95% CI: 1.22–1.76) and magnesium (OR = 1.24, 95% CI: 1.03–1.49) were associated with increased risk of UC.

**Conclusions:**

Our study provided evidence that circulating levels of antioxidants, minerals and vitamins might be causally linked to the development of IBD.

## Graphical abstract

Circulating levels of antioxidants, minerals and vitamins might be causally associated with the development of IBD.

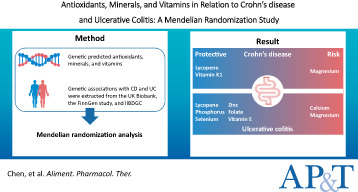

## 1 INTRODUCTION

Inflammatory bowel disease (IBD), including Crohn's Disease (CD) and ulcerative colitis (UC), is a chronic inflammatory disorder of the gastrointestinal tract with a rising incidence in populations.^1^ Although the precise aetiology of IBD remains unclear, accumulating evidence from observational studies suggests that dietary nutrients especially antioxidants, minerals and vitamins contribute to the pathogenesis of IBD.^2^, ^3^ Unfortunately, published research on the associations between dietary nutrients, especially antioxidants, minerals and vitamins and IBD is scarce and inconclusive. An umbrella review of meta‐analyses found that high vitamin D levels decreased the risk of CD and UC^4^; however, a randomised controlled trial (RCT) examining the effect of supplemental vitamin D did not show a significant clinical benefit.^5^ Large prospective cohort studies have reported that dietary zinc intake was inversely associated with incident CD,^6^, ^7^ whereas in a small RCT, zinc supplementation seemed to play little part in restraining inflammation in patients with IBD.^8^ Potential explanations for these inconsistent results may relate to the substantial biases (residual confounding and reverse causation) in observational studies as well as low adherence to treatment, low treatment doses, short trial duration and low statistical power in RCTs. The causal role of antioxidants, minerals and vitamins in the development of CD or UC remains unclear.

Mendelian randomization (MR) utilises genetic variants as instruments to make inferences in causal relationships between risk factors and disease outcomes.^9^ As germline genetic variants are randomly allocated at meiosis, MR design minimises confounding and is not influenced by environmental or self‐adopted factors and therefore strengthens causal inference. Construction of a validated MR association relies on three key assumptions: (1) genetic variant is associated with the exposure; (2) the genetic variant is not related to confounding; (3) the genetic variant has no effect on outcome directly.^10^ A previous MR study including 25,042 IBD cases indicated no association of genetically predicted vitamin D levels with CD or UC risk.^11^ Still, the MR associations of antioxidants, minerals and vitamins with CD or UC risk have not been systematically evaluated. Here, we conducted an MR investigation to comprehensively explore the causal associations of antioxidants, minerals and vitamins with CD and UC.

## 2 METHODS

### 2.1 Study design

**FIGURE 1:**
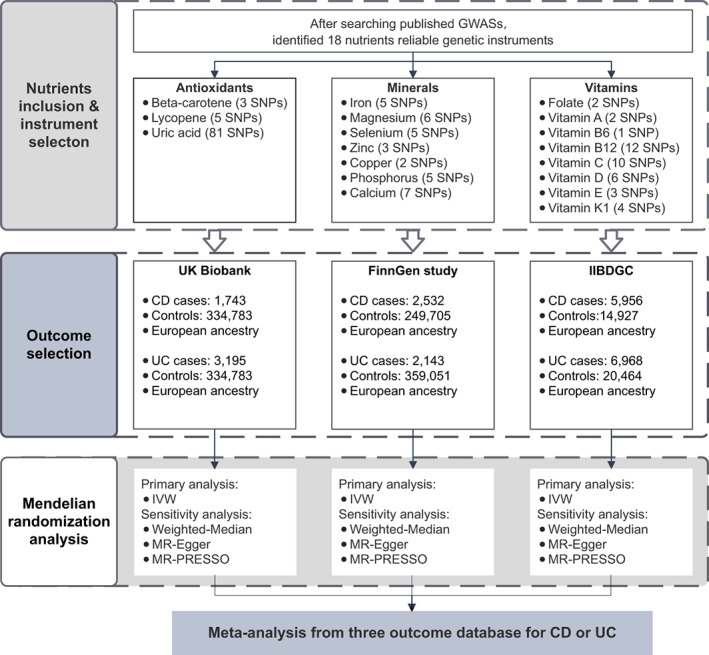
A flowchart of study design. CD, Crohn's disease; UC, ulcerative colitis; IIBDGC, The International Inflammatory Bowel Disease Genetics Consortium; IVW, inverse‐variance weighted; MR‐PRESSO, MR Pleiotropy RESidual Sum and Outlier.

This MR study design is depicted in Figure 1. The present study was based on publicly available data from UK biobank,^12^ the FinnGen study,^13^ the International Inflammatory Bowel Disease Genetics Consortium (IIBDGC)^14^ and published genome‐wide association studies (GWASs). All exposure‐specific MR analyses were conducted separately in UK Biobank, FinnGen study, and IIBDGC, and individual results were subsequently meta‐analysed to pool estimates for each exposure on CD or UC risk. Included studies had been approved by corresponding institutional review boards and ethical committees.

### 2.2 Instrumental variable selection

We conducted a search of the latest published GWASs performed among European individuals on diet‐related antioxidants, minerals and vitamins at the circulating level in the NHGRI‐EBI GWAS catalogue and PubMed (from inception to 1st March 2022). After the searching, published GWASs for 20 exposures were initially identified: beta‐carotene,^15^ lycopene,^16^ uric acid,^17^ calcium,^18^ copper,^19^ magnesium,^20^ sodium,^20^ potassium,^20^ copper,^19^ zinc,^19^ iron,^21^ selenium,^22^ phosphorus,^23^ folate,^24^ vitamins A,^25^ B6,^26^ B12,^24^ C,^27^ D,^28^ E^29^ and K1^30^ (Table S1). For some circulating nutrients such as sulphur and vitamin B1, no available GWASs were found, so these were not considered in the current study. Even though more recent GWASs on calcium, phosphorus and vitamin D were conducted in UK Biobank samples in which hundreds of SNPs were identified,^31^ we decided not to apply this option due to the overlap of the study population.

To comprehensively evaluate the effects of circulating antioxidants, minerals and vitamins on disease and obtain suitable instrumental variables (IVs), we selected eligible genetic instruments based on the following criteria: (1) SNPs should be associated with these circulating nutrients at a genome‐wide significance level (*p* < 5 × 10^−8^); for those traits instrumented by <2 SNPs, suggestive significant genome‐wide association significant (*p* < 1 × 10^−5^) or validated SNPs were included if available; (2) SNPs should be associated with exposure independently—that is, not in linkage disequilibrium (defined as *r* ^2^ < 0.01) with other genetic instruments for the same exposure; (3) the selected genetic instruments should explain at least 0.1% of the variance of exposure to ensure the strength of genetic IVs to be sufficient to evaluate a causal effect.^32^ Potassium and sodium were excluded because the criteria were not met and therefore 18 circulating nutrients were included in the analysis.

Detailed information of genetic instruments used for each nutrient is shown in Table S2. Notably, SNPs associated with circulating lycopene passing the threshold of *p* < 1 × 10^−6^ were identified from a small GWAS conducted in 441 individuals of European ancestry, explaining a substantial part (30.1%) of the total variance of circulating lycopene levels.^16^, ^33^ SNPs for vitamin K1 in five loci passing the threshold of *p* < 1 × 10^−6^ were reported in a GWAS of 2138 European individuals. Four SNPs with the strongest association with vitamin K1 levels in each locus were utilised explaining approximately 6.0% of genetic variance, and one SNP that was strongly associated with triglyceride and cholesterol was removed to minimise pleiotropy.^30^ The GWAS for lycopene, vitamins A, E and D levels were adjusted for body mass index. For each exposure, instrumental variables were harmonised to omit ambiguous SNPs with non‐concordant alleles and palindromic SNPs with ambiguous MAF (above 0.42 and <0.58). When an exposure‐associated SNP was not available in the outcome GWAS, a proxy SNP identified in high linkage disequilibrium (*r* ^2^ > 0.8) was used instead (using the LDlink tool and the European 1000 Genomes data^34^). The variance explained by genetic instruments for each nutrient ranged from 0.2% to 30.1% (Table S3).

### 2.3 Outcome data sources

Summary‐level GWAS data for CD and UC were available in UK Biobank,^12^ the FinnGen study,^13^ and IIBDGC.^14^ The UK Biobank study is a large multicenter cohort study that recruited more than 500,000 European participants across the United Kingdom between 2006 and 2010.^12^ In this study, summary statistics of genetic associations in UK Biobank were extracted from GWAS conducted by Lee lab.^35^ Crohn's disease (1743 cases and 334,783 controls) was defined according to the International Classification of Diseases 9th Revision (ICD‐9) (555) and ICD‐10 (K50); ulcerative Colitis (3195 cases and 334,783 controls) was defined according to ICD‐9 (556.9) and ICD‐10 (K51). The genetic‐disease association estimates were obtained by logistic regression with adjustment for the genetic principal components, sex and birth year. Summary‐level estimates of genetic associations with CD and UC were also obtained in the last publicly available R6 data release of the FinnGen study. The FinnGen study is a large nationwide cohort study launched in 2017, which combined genetic data from Finnish biobanks and digital health record data from Finnish health registries.^13^ A CD diagnosis (2532 cases and 249,705 controls) was coded according to the ICD‐8 (5630), ICD‐9 (555), ICD‐10 (K50); a UC diagnosis (5349 cases and 249,705 controls) was coded according to the ICD‐8 (5631, 569), ICD‐9 (556), ICD‐10 (K51). Genome‐wide association analyses for each trait were adjusted for sex, age, genetic components and genotyping batch. IIBDGC brings together genome‐wide genotyping data and whole‐genome sequencing data for over 75,000 patients with IBD.^14^ Diagnosis of IBD in IIBDGC was based on accepted radiologic, endoscopic and histopathologic evaluations. The genetic associations were obtained from logistic regression adjusted for age, sex and genetic principal components. We employed European ancestry summary‐level statistics including data for CD (5956 cases and 14,927 controls) and UC (6968 cases and 20,464 controls).

### 2.4 Statistical analysis

The primary MR analyses were conducted by utilising the inverse‐variance weighted method. For exposures with more than 3 SNPs, the estimates for variants were then pooled using the random‐multiplicative effects inverse‐variance weighted method. For exposures instrumented by only 2 SNPs, the fixed‐effects inverse‐variance weighted method was employed. The inverse‐variance weighted method provides the most precise estimates but assumes that all SNPs are valid instruments and any pleiotropy is balanced.^36^ Heterogeneity among estimates based on individual SNPs was assessed with Cochran's *Q* value. Wald ratio method was performed if there was only 1 SNP for the exposure, in which the SNP‐outcome association estimate was divided by its SNP‐exposure association estimate to obtain the causal relationship.

To examine if there was any violation of the assumptions of MR or any other potential biases for exposure with 3 or more genetic instruments, the weighted median,^36^ MR‐egger regression,^37^ and Mendelian randomization pleiotropy residual sum and outlier (MR‐PRESSO)^38^ methods were additionally performed as sensitivity analyses. The weighted median model can provide consistent estimates if at least 50% of the weight comes from valid instrumental variables.^36^ The intercept test of MR‐Egger regression can be used as an indicator of horizontal pleiotropy albeit with low, precise estimates.^37^ MR‐PRESSO method can identify horizontal pleiotropic outliers of SNPs and provide results identical to those from IVW in the absence of outliers.^38^

Fixed‐effects meta‐analysis was conducted to combine MR estimates from different data sources. The *F*‐statistic was estimated to quantify instrument strength for each exposure and an *F*‐statistic > 10 suggests a sufficiently strong instrument. We conducted power analysis by using the online web tool mRnd (https://cnsgenomics.shinyapps.io/mRnd/)^32^ (Table S3). The Benjamini–Hochberg correction that controls the false discovery rate (FDR) was applied to correct for the multiple testing separately for CD and UC, and associations with a Benjamini–Hochberg adjusted *p* < 0.05 were regarded as significant. All analyses were two‐sided and performed using the TwoSampleMR,^39^ and MRPRESSO R package^38^ in R software 4.1.2.

## 3 RESULTS

### 3.1 The causal role of antioxidants, minerals and vitamins in CD

**FIGURE 2:**
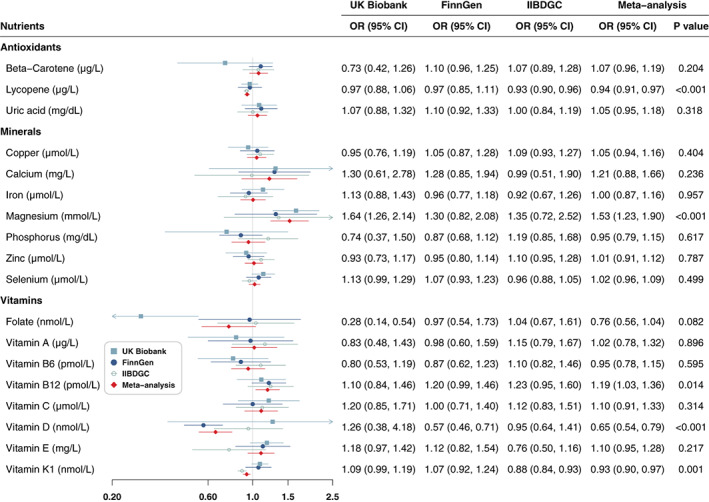
Associations of antioxidants, minerals and vitamins with risk of Crohn's disease. CI, confidence interval; OR, odds ratio.

Genetically predicted higher circulating lycopene levels were associated with reduced risk of CD in IIBDGC, and the associations were directionally consistent in the other two databases (Figure 2). In the meta‐analysis, the odds ratio (OR) of CD was 0.94 (95% CI: 0.91–0.97; *p* < 0.001) for a 1 μg/dl increase in genetically predicted lycopene (Table S4). Genetically predicted circulating magnesium levels were associated with CD risk in UK Biobank, and the associations were directionally consistent in the other two databases (Figure 2). For one SD increase in genetically predicted circulating magnesium levels, the combined OR of CD was 1.53 (95% CI: 1.23–1.90; *p* < 0.001). Genetically predicted vitamin D (OR = 0.65, 95% CI: 0.54–0.79; *p* < 0.001, per SD increase) and vitamin K1 (OR = 0.93, 95% CI: 0.90–0.97; *p* = 0.001, per 1‐unit increase in natural logarithm‐transformed) levels were inversely associated with the disease in the meta‐analyses (Figure 2).

Associations across these sensitivity analyses were generally directionally consistent. Heterogeneity was detected in the analysis of magnesium, vitamins B12 and D (Table S5). The MR‐Egger regression intercept suggested evidence of horizontal pleiotropy in the analysis of magnesium in IIBDGC. A few outliers were detected in the MR‐PRESSO analysis of magnesium and vitamin B12, and the removal of these outliers did not change the direction of the associations.

### 3.2 The causal role of antioxidants, minerals and vitamins in UC

**FIGURE 3:**
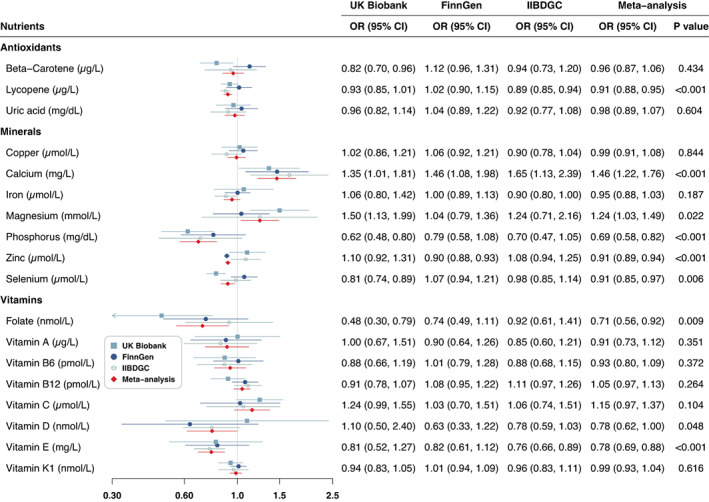
Associations of antioxidants, minerals and vitamins with risk of ulcerative colitis. CI, confidence interval; OR, odds ratio.

Genetically predicted higher lycopene levels were associated with reduced risk of UC in IIBDGC, and the associations were directionally consistent in UK Biobank (Figure 3). For a 1 μg/dl increase in the genetically predicted lycopene levels, the combined OR of UC was 0.91 (95% CI: 0.88–0.95; *p* < 0.001). Higher genetically predicted phosphorus, zinc and selenium levels were statistically associated with a decreased risk of UC, whereas genetically predicted calcium and magnesium levels were positively associated with the disease (Figure 3). The combined ORs per 1‐SD increase in genetically predicted circulating levels of these minerals were 0.69 (95% CI: 0.58–0.82; *p* < 0.004) for phosphorus, 0.91 (95% CI: 0.85–0.97; *p* = 0.006) for selenium, 1.46 (95% CI: 1.22–1.76; *p* = 0.022) for calcium and 1.24 (95% CI: 1.03–1.49; *p* = 0.022) for magnesium. Genetically predicted folate (OR = 0.71, 95% CI: 0.56–0.92; *p* = 0.009, per SD increase) and vitamin E (OR = 0.78, 95% CI: 0.69–0.88; *p* < 0.001) levels were inversely associated with risk of UC in the meta‐analyses (Figure 3; Table S4).

Results from the sensitivity analyses were generally consistent with the primary analysis, though they did not always reach a significant level. Associations between SNPs on magnesium and risk of UC showed evidence of heterogeneity in IIBDGC, and the direction of the association did not alter after removing one outlier in MR‐PRESSO analysis (Table S6).

## 4 DISCUSSION

In this MR analysis, we provided evidence that genetically predicted higher circulating lycopene, vitamins D and K1 levels were associated with reduced risk of CD, whereas the genetically predicted high magnesium levels were associated with elevated CD risk. Our study also found that genetically predicted circulating levels of lycopene, phosphorus, selenium, zinc, folate and vitamin E were inversely associated with UC risk, and genetically predicted higher calcium and magnesium levels were associated with increased risk of UC.

The protective role of antioxidants on IBD risk has been explored in previous observational studies.^40^, ^41^, ^42^ An inverse association of lycopene levels with CD and UC was detected in our MR analysis, which was in line with previous studies.^40^, ^41^ Results from a cross‐sectional study with 37 nonsmoking CD patients showed that plasma lycopene was significantly lower in CD patients than in controls^40^; another cross‐sectional study of 56 UC patients in remission showed that higher lycopene intakes were associated with lower faecal blood, mucus and pus among participants.^42^ Evidence from laboratory studies indicated several underlying mechanisms. Lycopene is one of the free radical scavengers, which neutralises free radicals by donating electrons^43^ and thus delays the process of oxidative stress in IBD. A recent experimental animal study demonstrated that lycopene plays a preventive role in dextran sulphate sodium‐induced colitis mice by regulating the TLR4/TRIF/NF‐κB signalling pathway.^44^ Published studies also examined the associations between circulating levels of β‐carotene, retinol and ascorbate and the risk of CD and UC, but reported inconsistent findings.^40^, ^42^, ^45^ In this MR investigation, we observed limited evidence in support of causal associations for genetically predicted beta‐carotene, retinol (vitamin A) and ascorbate (vitamin C) in relation to IBD risk. The discrepancy between the observational studies and our MR study might be attributed to the residual confounding and reverse causation bias, or insufficient statistical power. In the case of lycopene, limited studies made the supplementation recommendations vague and dispensable. Taken together, our study supports that lycopene is one of the promising antioxidants with the potential for reducing both CD and UC risk.

Data on the associations of minerals with IBD are scarce. Inconsistent with our results, most cross‐sectional studies observed that circulating calcium levels were lower in IBD patients.^46^, ^47^ A most likely explanation for this discrepancy is that gastrointestinal damage as a result of ongoing inflammation leads to the malabsorption of calcium after the onset of IBD. Interestingly, we observed that genetic predisposition to higher circulating calcium and genetic predisposition to lower circulating phosphorus was associated with elevated UC risk with validated genetic instruments, which indicated that the disorders of calcium and phosphorus metabolism may be involved in the pathological process of UC onset. Considering the low variance explained by the genetic instrument for calcium (0.8%) and phosphorus (0.2%), we cannot rule out that weak associations between these two minerals and CD were overlooked. Although the mechanistic explanations for the association between calcium and UC remain unclear, it has been reported that serum calcium might contribute to the inflammation by activating the inflammasome through the calcium‐sensing receptor.^48^ Besides, animal models have provided evidence that calcium/calmodulin‐dependent protein kinase IV activation contributes to the pathogenesis of experimental colitis via inhibition of intestinal epithelial cell proliferation.^49^

We also observed that genetic predisposition to higher zinc and selenium levels were associated with a decreased risk of UC. Evidence from two large prospective cohorts of women based on semi‐quantitative food frequency questionnaires showed that intake of zinc was inversely associated with risk of CD but not UC.^6^ Another prospective cohort based on 24‐hour dietary records reported that dietary zinc intake was inversely associated with incident CD.^7^ We found a statistically significant positive association of circulating magnesium with CD and UC, which are novel and therefore require confirmation in further studies. Several biological mechanisms have been suggested for explaining the protective roles of several minerals, such as the anti‐inflammatory effect mediated by selenium^50^ and the regulation of immune function by zinc.^51^ As for magnesium, a previous MR study uncovered that genetically predicted magnesium is associated with an 8.74‐fold increased risk of rheumatoid arthritis^52^; however, definitive proof for mechanisms of magnesium in the regulation of immune diseases is still lacking.

The role of vitamin D in CD risk has been noted previously. A prospective cohort study with 122 incident cases of CD and 123 cases of UC in the Nurses' Health Study found that higher predicted plasma levels of 25(OH)D were associated with a 46% lower risk of incident CD.^53^ A previous MR study on vitamin D including approximately 2000 IBD cases found no causal association with either CD or UC risk.^54^ Another MR study including 25,042 IBD cases of mixed ancestry, provided limited evidence for the association of vitamin D with CD.^11^ On the contrary, our MR study identified significant MR associations between vitamin D levels and the risk of CD and UC. These conflicting findings might be caused by the enhanced power of the current MR study, in which we used 3 databases to estimate the association with greater precision. In addition, the reliability of the results was significantly improved due to an increasing number of genetic instruments. We also observed the inverse association between vitamin K1 and CD. It has been reported that circulating vitamin K was insufficient in patients with CD, which was suggested to be associated with inflammatory processes.^55^ Lacking prospective evidence, more studies are warranted to confirm this observed association. We also observed inverse associations of circulating folate and vitamin E with UC risk. In line with our results, a meta‐analysis including 12 studies indicated that the average serum folate concentration in patients with UC was significantly lower than that in controls.^56^ The inverse association between vitamin E and UC has been uncovered in some previous observational studies^57^, ^58^ but not all.^59^ Detailed pathophysiological mechanisms behind the association between vitamins and IBD remained elusive but several hypotheses are supporting such relationships. It is recognised that vitamin D is an essential anti‐inflammatory nutrient in regulating gut mucosal immunity.^60^ Studies suggested that vitamin D may affect intestinal epithelium integrity and innate immune barrier function in the involvement of IBD.^60^ Emerging studies support that vitamin K1 is involved in immune response and anti‐inflammation^61^ and is associated with the protective and promoting role in the intestine.^62^ It has been proved that folate plays a role in regulating reactive oxygen species production and reducing oxidative stress by acting directly as an antioxidant.^63^ Vitamin E reduces lipid peroxidation and thus has a significant role in membrane stabilization^64^ which may mitigate the oxidative stress in UC. The different vitamins in relation to CD and UC highlight distinctive pathological pathways in UC and CD. The majority of the literature on vitamin deficiencies in IBD centres around deficiencies in folate, vitamins D, B12, E and K were relatively overlooked. Deficiencies on vitamins E and K were usually underappreciated in IBD patients with steatorrhea and the pre‐morbid state was easily neglected.^65^ Findings on protective role of vitamins E and K1 provided new insight into the pathogenesis of UC and CD.

Current European Society for Clinical Nutrition and Metabolism (ESPEN) recommendations suggest that micronutrient levels need to be assessed annually in IBD patients, with the correction of any deficit with supplementation.^66^ Similarly, literature on supplementation of other micronutrients as therapy is limited to a few, small‐scale studies that only target specific subgroups of patients. Limited evidence makes specific recommendations on nutrient supplementation difficult. Findings from this study will complement the current evidence from observational studies supporting that circulating micronutrients of antioxidants, minerals and vitamins are causally linked to the development of IBD, which would contribute to the research field of nutrient prevention for IBD.

The main strength of this study is the MR design that incorporated data from large consortia to provide solid genetic evidence for the reported associations. The MR study with data from populations of European ancestry reduces the risk of confounding and reverse causality and also minimises bias caused by population stratification. In addition, three large databases with independent populations comprising 10,231 CD and 15,383 UC cases for gene‐outcome associations were meta‐analysed in the present study. The results from these three data sources were generally consistent, making it less likely that the observed associations were caused by chance.

Limitations of this MR investigation also merit consideration. Genetic instruments used in this study collectively explained only a small proportion of the variance in blood levels of calcium, phosphorus and folate, which may result in insufficient power to detect small or moderate associations. However, the large sample sizes in our outcomes datasets also alleviate the relatively inadequate statistical power. In addition, the small sample size of GWAS in lycopene, zinc and vitamin B6 may contribute to imprecision in the selection of SNPs. Therefore, there is a need for larger GWAS to identify more genetic variants for nutrients. Another limitation in MR analysis is horizontal pleiotropy, but there was no indication of pleiotropic effects in MR‐Egger analyses. A further limitation is that the relationship among nutrients is complex, and it may be misleading to examine nutrients individually without considering others. Although some endogenous nutrients are related to exogenous dietary intake in dose‐dependently manners, changes in circulating levels of nutrients do not necessarily and completely reflect the variations in dietary intake of nutrients. Besides, genetic associations with lycopene, vitamins A, D and E were estimated while adjusted for body mass index according to the original GWAS, which might increase the risk for collider bias in the current MR investigation. Unfortunately, the GWASs for these nutrients without adjustment for BMI were not available, thus these observed associations should be cautiously interpreted.

## 5 CONCLUSION

In conclusion, this MR study observed that genetically predicted lycopene, vitamins D and K1 levels were inversely associated with CD risk, whereas the genetically predicted magnesium levels were positively associated with CD risk. We also found evidence that genetically predicted lycopene, phosphorus, selenium, zinc, folate and vitamin E levels have an inverse effect on the risk of UC, and genetic prediction of high calcium and magnesium levels was associated with increased risk of UC.

## AUTHORSHIP

*Guarantor of the article*: Xiaoyan Wang and Xue Li.

*Author contributions*: All authors made critical revisions of the manuscript for important intellectual content. All authors have read and approved the final version of the manuscript. Jie Chen (Conceptualization: Equal; Methodology: Equal; Formal analysis: Equal; Data curation: Equal; and Writing – original draft: Equal). Xixian Ruan (Conceptualization: Equal; Methodology: Equal; Formal analysis: Equal; and Writing – original draft: Equal). Shuai Yuan (Conceptualization: Supporting; Methodology: Supporting; Writing – review & editing: Supporting). Minzi Deng (Conceptualization: Supporting; Writing – review & editing: Supporting). Han Zhang (Conceptualization: Supporting; Writing – review & editing: Supporting). Jing Sun (Conceptualization: Supporting; Writing – review & editing: Supporting). Lili Yu (Conceptualization: Supporting; Writing – review & editing: Supporting). Jack Satsangi (Conceptualization: Supporting; Methodology: Supporting; Writing – review & editing: Supporting). Susanna C. Larsson (Conceptualization: Equal; Methodology: Equal; Data curation: Equal; and Writing – review & editing: Leading). Evropi Therdoratou (Conceptualization: Equal; Methodology: Equal; Writing – review & editing: Leading). Xiaoyan Wang (Conceptualization: Leading; Data curation: Equal; and Funding acquisition: Leading; Writing – review & editing: Equal). Xue Li (Conceptualization: Equal; Data curation: Equal; Funding acquisition: Equal; and Writing – review & editing: Leading). All authors approved the final version of the manuscript.

## FUNDING INFORMATION

XL is supported by the Natural Science Fund for Distinguished Young Scholars of Zhejiang Province (LR22H260001). ET is supported by a CRUK Career Development Fellowship (C31250/A22804). SCL acknowledges research funding from the Swedish Heart Lung Foundation (Hjärt‐Lungfonden, 20,210,351), the Swedish Research Council (Vetenskapsrådet, 2019‐00977) and the Swedish Cancer Society (Cancerfonden). XYW is supported by National Natural Science Foundation of China (81970494) and Key Project of Research and Development Plan of Hunan Province (2019SK2041).

## CONFLICT OF INTEREST

All authors declare no competing interest.

## Supporting information


Table S1‐S6.

## References

[1] Dahlhamer JM , Zammitti EP , Ward BW , Wheaton AG , Croft JB . Prevalence of inflammatory bowel disease among adults aged >/=18 years – United States, 2015. MMWR Morb Mortal Wkly Rep. 2016;65(42):1166–9. 10.15585/mmwr.mm6542a3 27787492

[2] Hou JK , Abraham B , El‐Serag H . Dietary intake and risk of developing inflammatory bowel disease: a systematic review of the literature. Am J Gastroenterol. 2011;106(4):563–73. 10.1038/ajg.2011.44 21468064

[3] Lee D , Albenberg L , Compher C , Baldassano R , Piccoli D , Lewis JD , et al. Diet in the pathogenesis and treatment of inflammatory bowel diseases. Gastroenterology. 2015;148(6):1087–106. 10.1053/j.gastro.2015.01.007 25597840 PMC4409494

[4] Piovani D , Danese S , Peyrin‐Biroulet L , Nikolopoulos GK , Lytras T , Bonovas S . Environmental risk factors for inflammatory bowel diseases: an umbrella review of meta‐analyses. Gastroenterology. 2019;157(3):647–659 e4. 10.1053/j.gastro.2019.04.016 31014995

[5] Guo Y , Zhang T , Wang Y , Liu R , Chang M , Wang X . Effects of oral vitamin D supplementation on inflammatory bowel disease: a systematic review and meta‐analysis. Food Funct. 2021;12(17):7588–606. 10.1039/d1fo00613d 34231596

[6] Ananthakrishnan AN , Khalili H , Song M , Higuchi LM , Richter JM , Chan AT . Zinc intake and risk of Crohn's disease and ulcerative colitis: a prospective cohort study. Int J Epidemiol. 2015;44(6):1995–2005. 10.1093/ije/dyv301 26546032 PMC5156337

[7] Vasseur P , Dugelay E , Benamouzig R , Savoye G , Hercberg S , Touvier M , et al. Dietary zinc intake and inflammatory bowel disease in the French NutriNet‐Sante cohort. Am J Gastroenterol. 2020;115(8):1293–7. 10.14309/ajg.0000000000000688 32467505

[8] Mulder TP , van der Sluys VA , Verspaget HW , Griffioen G , Peña AS , Janssens AR , et al. Effect of oral zinc supplementation on metallothionein and superoxide dismutase concentrations in patients with inflammatory bowel disease. J Gastroenterol Hepatol. 1994;9(5):472–7. 10.1111/j.1440-1746.1994.tb01277.x 7827298

[9] Davey Smith G , Hemani G . Mendelian randomization: genetic anchors for causal inference in epidemiological studies. Hum Mol Genet. 2014;23(R1):R89–98. 10.1093/hmg/ddu328 25064373 PMC4170722

[10] Burgess S , Davey Smith G , Davies NM , Dudbridge F , Gill D , Glymour MM , et al. Guidelines for performing Mendelian randomization investigations. Wellcome Open Res. 2019;4:186. 10.12688/wellcomeopenres.15555.2 32760811 PMC7384151

[11] Carreras‐Torres R , Ibanez‐Sanz G , Obon‐Santacana M , Duell EJ , Moreno V . Identifying environmental risk factors for inflammatory bowel diseases: a Mendelian randomization study. Sci Rep. 2020;10(1):19273. 10.1038/s41598-020-76361-2 33159156 PMC7648100

[12] Sudlow C , Gallacher J , Allen N , Beral V , Burton P , Danesh J , et al. UK biobank: an open access resource for identifying the causes of a wide range of complex diseases of middle and old age. PLoS Med. 2015;12(3):e1001779. 10.1371/journal.pmed.1001779 25826379 PMC4380465

[13] FinnGen . FinnGen Documentation of R6 release. https://finngen.gitbook.io/documentation/

[14] Liu JZ , van Sommeren S , Huang H , Ng SC , Alberts R , Takahashi A , et al. Association analyses identify 38 susceptibility loci for inflammatory bowel disease and highlight shared genetic risk across populations. Nat Genet. 2015;47(9):979–86. 10.1038/ng.3359 26192919 PMC4881818

[15] Hendrickson SJ , Hazra A , Chen C , Eliassen AH , Kraft P , Rosner BA , et al. Beta‐carotene 15,15′‐monooxygenase 1 single nucleotide polymorphisms in relation to plasma carotenoid and retinol concentrations in women of European descent. Am J Clin Nutr. 2012;96(6):1379–89. 10.3945/ajcn.112.034934 23134893 PMC3497927

[16] D'Adamo CR , D'Urso A , Ryan KA , Yerges‐Armstrong LM , Semba RD , Steinle NI , et al. A common variant in the SETD7 gene predicts serum lycopene concentrations. Nutrients. 2016;8(2):82. 10.3390/nu8020082 26861389 PMC4772045

[17] Tin A , Marten J , Halperin Kuhns VL , Li Y , Wuttke M , Kirsten H , et al. Target genes, variants, tissues and transcriptional pathways influencing human serum urate levels. Nat Genet. 2019;51(10):1459–74. 10.1038/s41588-019-0504-x 31578528 PMC6858555

[18] O'Seaghdha CM , Wu H , Yang Q , Kapur K , Guessous I , Zuber AM , et al. Meta‐analysis of genome‐wide association studies identifies six new loci for serum calcium concentrations. PLoS Genet. 2013;9(9):e1003796. 10.1371/journal.pgen.1003796 24068962 PMC3778004

[19] Evans DM , Zhu G , Dy V , Heath AC , Madden PAF , Kemp JP , et al. Genome‐wide association study identifies loci affecting blood copper, selenium and zinc. Hum Mol Genet. 2013;22(19):3998–4006. 10.1093/hmg/ddt239 23720494 PMC3766178

[20] Meyer TE , Verwoert GC , Hwang SJ , Glazer NL , Smith AV , van Rooij FJA , et al. Genome‐wide association studies of serum magnesium, potassium, and sodium concentrations identify six loci influencing serum magnesium levels. PLoS Genet. 2010;6(8):e1001045. 10.1371/journal.pgen.1001045 20700443 PMC2916845

[21] Benyamin B , Esko T , Ried JS , Radhakrishnan A , Vermeulen SH , Traglia M , et al. Novel loci affecting iron homeostasis and their effects in individuals at risk for hemochromatosis. Nat Commun. 2014;5:4926. 10.1038/ncomms5926 25352340 PMC4215164

[22] Cornelis MC , Fornage M , Foy M , Xun P , Gladyshev VN , Morris S , et al. Genome‐wide association study of selenium concentrations. Hum Mol Genet. 2015;24(5):1469–77. 10.1093/hmg/ddu546 25343990 PMC4321444

[23] Kestenbaum B , Glazer NL , Kottgen A , Felix JF , Hwang SJ , Liu Y , et al. Common genetic variants associate with serum phosphorus concentration. J Am Soc Nephrol. 2010;21(7):1223–32. 10.1681/ASN.2009111104 20558539 PMC3152230

[24] Grarup N , Sulem P , Sandholt CH , Thorleifsson G , Ahluwalia TS , Steinthorsdottir V , et al. Genetic architecture of vitamin B12 and folate levels uncovered applying deeply sequenced large datasets. PLoS Genet. 2013;9(6):e1003530. 10.1371/journal.pgen.1003530 23754956 PMC3674994

[25] Mondul AM , Yu K , Wheeler W , Zhang H , Weinstein SJ , Major JM , et al. Genome‐wide association study of circulating retinol levels. Hum Mol Genet. 2011;20(23):4724–31. 10.1093/hmg/ddr387 21878437 PMC3209826

[26] Tanaka T , Scheet P , Giusti B , Bandinelli S , Piras MG , Usala G , et al. Genome‐wide association study of vitamin B6, vitamin B12, folate, and homocysteine blood concentrations. Am J Hum Genet. 2009;84(4):477–82. 10.1016/j.ajhg.2009.02.011 19303062 PMC2667971

[27] Zheng JS , Luan J , Sofianopoulou E , Imamura F , Stewart ID , Day FR , et al. Plasma vitamin C and type 2 diabetes: genome‐wide association study and Mendelian randomization analysis in European populations. Diabetes Care. 2021;44(1):98–106. 10.2337/dc20-1328 33203707 PMC7783939

[28] Jiang X , O'Reilly PF , Aschard H , Hsu YH , Richards JB , Dupuis J , et al. Genome‐wide association study in 79,366 European‐ancestry individuals informs the genetic architecture of 25‐hydroxyvitamin D levels. Nat Commun. 2018;9(1):260. 10.1038/s41467-017-02662-2 29343764 PMC5772647

[29] Major JM , Yu K , Wheeler W , Zhang H , Cornelis MC , Wright ME , et al. Genome‐wide association study identifies common variants associated with circulating vitamin E levels. Hum Mol Genet. 2011;20(19):3876–83. 10.1093/hmg/ddr296 21729881 PMC3168288

[30] Dashti HS , Shea MK , Smith CE , Tanaka T , Hruby A , Richardson K , et al. Meta‐analysis of genome‐wide association studies for circulating phylloquinone concentrations. Am J Clin Nutr. 2014;100(6):1462–9. 10.3945/ajcn.114.093146 25411281 PMC4232014

[31] Sinnott‐Armstrong N , Tanigawa Y , Amar D , Mars N , Benner C , Aguirre M , et al. Genetics of 35 blood and urine biomarkers in the UK biobank. Nat Genet. 2021;53(2):185–94. 10.1038/s41588-020-00757-z 33462484 PMC7867639

[32] Brion MJ , Shakhbazov K , Visscher PM . Calculating statistical power in Mendelian randomization studies. Int J Epidemiol. 2013;42(5):1497–501. 10.1093/ije/dyt179 24159078 PMC3807619

[33] Luo J , le Cessie S , van Heemst D , Noordam R . Diet‐derived circulating antioxidants and risk of coronary heart disease: a Mendelian randomization study. J Am Coll Cardiol. 2021;77(1):45–54. 10.1016/j.jacc.2020.10.048 33413940

[34] Machiela MJ , Chanock SJ . LDlink: a web‐based application for exploring population‐specific haplotype structure and linking correlated alleles of possible functional variants. Bioinformatics. 2015;31(21):3555–7. 10.1093/bioinformatics/btv402 26139635 PMC4626747

[35] Zhou W , Zhao Z , Nielsen JB , Fritsche LG , LeFaive J , Gagliano Taliun SA , et al. Scalable generalized linear mixed model for region‐based association tests in large biobanks and cohorts. Nat Genet. 2020;52(6):634–9. 10.1038/s41588-020-0621-6 32424355 PMC7871731

[36] Yavorska OO , Burgess S . MendelianRandomization: an R package for performing Mendelian randomization analyses using summarized data. Int J Epidemiol. 2017;46(6):1734–9. 10.1093/ije/dyx034 28398548 PMC5510723

[37] Burgess S , Thompson SG . Interpreting findings from Mendelian randomization using the MR‐egger method. Eur J Epidemiol. 2017;32(5):377–89. 10.1007/s10654-017-0255-x 28527048 PMC5506233

[38] Verbanck M , Chen CY , Neale B , Do R . Detection of widespread horizontal pleiotropy in causal relationships inferred from Mendelian randomization between complex traits and diseases. Nat Genet. 2018;50(5):693–8. 10.1038/s41588-018-0099-7 29686387 PMC6083837

[39] Hemani G , Zheng J , Elsworth B , Wade KH , Haberland V , Baird D , et al. The MR‐Base platform supports systematic causal inference across the human phenome. eLife. 2018;7:e34408. 10.7554/eLife.34408 29846171 PMC5976434

[40] Wendland BE , Aghdassi E , Tam C , Carrrier J , Steinhart AH , Wolman SL , et al. Lipid peroxidation and plasma antioxidant micronutrients in Crohn disease. Am J Clin Nutr. 2001;74(2):259–64. 10.1093/ajcn/74.2.259 11470730

[41] D'Odorico A , Bortolan S , Cardin R , D'Inca' R , Martines D , Ferronato A , et al. Reduced plasma antioxidant concentrations and increased oxidative DNA damage in inflammatory bowel disease. Scand J Gastroenterol. 2001;36(12):1289–94. 10.1080/003655201317097146 11761019

[42] Glabska D , Guzek D , Zakrzewska P , Wlodarek D , Lech G . Lycopene, lutein and zeaxanthin may reduce faecal blood, mucus and pus but not abdominal pain in individuals with ulcerative colitis. Nutrients. 2016;8(10):613. 10.3390/nu8100613 27706028 PMC5084001

[43] Nimse SB , Pal D . Free radicals, natural antioxidants, and their reaction mechanisms. RSC Adv. 2015;5(35):27986–8006.

[44] Li Y , Pan X , Yin M , Li C , Han L . Preventive effect of lycopene in dextran sulfate sodium‐induced ulcerative colitis mice through the regulation of TLR4/TRIF/NF‐kappaB signaling pathway and tight junctions. J Agric Food Chem. 2021;69(45):13500–9. 10.1021/acs.jafc.1c05128 34729976

[45] Miyake Y , Tanaka K , Nagata C , Furukawa S , Andoh A , Yokoyama T , et al. Dietary intake of vegetables, fruit, and antioxidants and risk of ulcerative colitis: a case‐control study in Japan. Nutrition. 2021;91‐92:111378. 10.1016/j.nut.2021.111378 34265581

[46] Jasielska M , Grzybowska‐Chlebowczyk U . Hypocalcemia and vitamin D deficiency in children with inflammatory bowel diseases and lactose intolerance. Nutrients. 2021;13(8):2583. 10.3390/nu13082583 34444743 PMC8400662

[47] Sledzinska K , Landowski P , Zmijewski MA , Kaminska B , Kowalski K , Liberek A . Diet, sun, physical activity and vitamin D status in children with inflammatory bowel disease. Nutrients. 2022;14(5):1029. 10.3390/nu14051029 35268001 PMC8912613

[48] Jager E , Murthy S , Schmidt C , Hahn M , Strobel S , Peters A , et al. Calcium‐sensing receptor‐mediated NLRP3 inflammasome response to calciprotein particles drives inflammation in rheumatoid arthritis. Nat Commun. 2020;11(1):4243. 10.1038/s41467-020-17749-6 32843625 PMC7447633

[49] Cunningham KE , Novak EA , Vincent G , Siow VS , Griffith BD , Ranganathan S , et al. Calcium/calmodulin‐dependent protein kinase IV (CaMKIV) activation contributes to the pathogenesis of experimental colitis via inhibition of intestinal epithelial cell proliferation. FASEB J. 2019;33(1):1330–46. 10.1096/fj.201800535R 30113881 PMC6355073

[50] Krehl S , Loewinger M , Florian S , Kipp AP , Banning A , Wessjohann LA , et al. Glutathione peroxidase‐2 and selenium decreased inflammation and tumors in a mouse model of inflammation‐associated carcinogenesis whereas sulforaphane effects differed with selenium supply. Carcinogenesis. 2012;33(3):620–8. 10.1093/carcin/bgr288 22180572 PMC3291858

[51] Mayer LS , Uciechowski P , Meyer S , Schwerdtle T , Rink L , Haase H . Differential impact of zinc deficiency on phagocytosis, oxidative burst, and production of pro‐inflammatory cytokines by human monocytes. Metallomics. 2014;6(7):1288–95. 10.1039/c4mt00051j 24823619

[52] Cheng WW , Zhu Q , Zhang HY . Mineral nutrition and the risk of chronic diseases: a Mendelian randomization study. Nutrients. 2019;11(2):378. 10.3390/nu11020378 30759836 PMC6412267

[53] Ananthakrishnan AN , Khalili H , Higuchi LM , Bao Y , Korzenik JR , Giovannucci EL , et al. Higher predicted vitamin D status is associated with reduced risk of Crohn's disease. Gastroenterology. 2012;142(3):482–9. 10.1053/j.gastro.2011.11.040 22155183 PMC3367959

[54] Lund‐Nielsen J , Vedel‐Krogh S , Kobylecki CJ , Brynskov J , Afzal S , Nordestgaard BG . Vitamin D and inflammatory bowel disease: Mendelian randomization analyses in the Copenhagen studies and UK Biobank. J Clin Endocrinol Metab. 2018;103(9):3267–77. 10.1210/jc.2018-00250 29947775

[55] Nakajima S , Iijima H , Egawa S , Shinzaki S , Kondo J , Inoue T , et al. Association of vitamin K deficiency with bone metabolism and clinical disease activity in inflammatory bowel disease. Nutrition. 2011;27(10):1023–8. 10.1016/j.nut.2010.10.021 21482072

[56] Pan Y , Liu Y , Guo H , Jabir MS , Liu X , Cui W , et al. Associations between folate and vitamin B12 levels and inflammatory bowel disease: a meta‐analysis. Nutrients. 2017;9(4):382. 10.3390/nu9040382 28406440 PMC5409721

[57] Sampietro GM , Cristaldi M , Cervato G , Maconi G , Danelli P , Cervellione R , et al. Oxidative stress, vitamin a and vitamin E behaviour in patients submitted to conservative surgery for complicated Crohn's disease. Dig Liver Dis. 2002;34(10):696–701. 10.1016/s1590-8658(02)80020-2 12469796

[58] Levy E , Rizwan Y , Thibault L , Lepage G , Brunet S , Bouthillier L , et al. Altered lipid profile, lipoprotein composition, and oxidant and antioxidant status in pediatric Crohn disease. Am J Clin Nutr. 2000;71(3):807–15. 10.1093/ajcn/71.3.807 10702177

[59] Hengstermann S , Valentini L , Schaper L , Buning C , Koernicke T , Maritschnegg M , et al. Altered status of antioxidant vitamins and fatty acids in patients with inactive inflammatory bowel disease. Clin Nutr. 2008;27(4):571–8. 10.1016/j.clnu.2008.01.007 18316141

[60] Fletcher J , Cooper SC , Ghosh S , Hewison M . The role of vitamin D in inflammatory bowel disease: mechanism to management. Nutrients. 2019;11(5):1019. 10.3390/nu11051019 31067701 PMC6566188

[61] Kiely A , Ferland G , Ouliass B , O'Toole PW , Purtill H , O'Connor EM . Vitamin K status and inflammation are associated with cognition in older Irish adults. Nutr Neurosci. 2020;23(8):591–9. 10.1080/1028415X.2018.1536411 30451602

[62] Kieronska‐Rudek A , Kij A , Kaczara P , Tworzydlo A , Napiorkowski M , Sidoryk K , et al. Exogenous vitamins K exert anti‐inflammatory effects dissociated from their role as substrates for synthesis of endogenous MK‐4 in murine macrophages cell line. Cells. 2021;10(7):1571. 10.3390/cells10071571 34206530 PMC8303864

[63] Rezk BM , Haenen GR , van der Vijgh WJ , Bast A . Tetrahydrofolate and 5‐methyltetrahydrofolate are folates with high antioxidant activity. Identification of the antioxidant pharmacophore. FEBS Lett. 2003;555(3):601–5. 10.1016/s0014-5793(03)01358-9 14675781

[64] Miyazawa T , Burdeos GC , Itaya M , Nakagawa K , Miyazawa T . Vitamin E: regulatory redox interactions. IUBMB Life. 2019;71(4):430–41. 10.1002/iub.2008 30681767

[65] Gold SL , Manning L , Kohler D , Ungaro R , Sands B , Raman M . Micronutrients and their role in inflammatory bowel disease: function, assessment, supplementation, and impact on clinical outcomes including muscle health. Inflamm Bowel Dis. 2022;izac223. 10.1093/ibd/izac223 36287025

[66] Bischoff SC , Escher J , Hebuterne X , Kłęk S , Krznaric Z , Schneider S , et al. ESPEN practical guideline: clinical nutrition in inflammatory bowel disease. Clin Nutr. 2020;39(3):632–53. 10.1016/j.clnu.2019.11.002 32029281

