# Supplementary material for: Antioxidants, minerals and vitamins in relation to Crohn's disease and ulcerative colitis: A Mendelian randomization study
Source: Aliment Pharmacol Ther. 2023 Jan 16;57(4):399–408. doi: 10.1111/apt.17392 (PMC11497233; doi:10.1111/apt.17392)

Supplementary Tables for

**Antioxidants, Minerals, and Vitamins in Relation to Crohn’s disease and Ulcerative Colitis: A Mendelian Randomization Study**

Jie Chen, Xixian Ruan, Shuai Yuan, Minzi Deng, Han Zhang, Jing Sun, Lili Yu, Jack Satsangi, Susanna C. Larsson, Evropi Therdoratou, Xiaoyan Wang, Xue Li

| Table S1. Characteristics of used studies and consortia |
| --- |
| Table S2. Summary information for SNPs used as genetic instruments for Mendelian randomization analyses |
| Table S3. Power estimation of this Mendelian randomization analysis |
| Table S4. False discovery rate adjusted p values for all tested associations in meta-analysis of three data sources |
| Table S5. Associations of amino acids, antioxidants, minerals and vitamins with CD in sensitivity analyses |
| Table S6. Associations of amino acids, antioxidants, minerals and vitamins with UC in sensitivity analyses |

All tables can be obtained in OSF data respiratory:

<https://osf.io/42c5h/?view_only=00c696e11cb542bb837a2b14eec09c9d>

Please view the file by clicking the xlsx file.


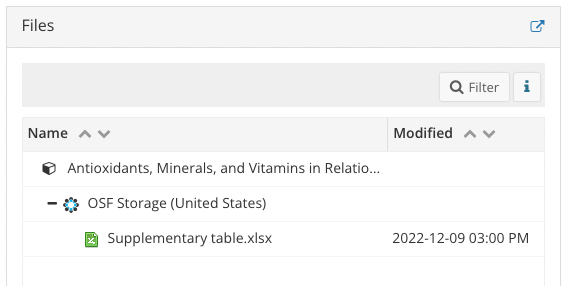

Supplement: Supplementary file 1 — Table S1‐S6. [file APT-57-399-s001.docx]
